# The effect of influenza vaccination on the rate of dementia amongst older adults

**DOI:** 10.1111/ene.16489

**Published:** 2024-10-06

**Authors:** Andreas Moses Appel, Janet Janbek, Christina Jensen‐Dahm, Thomas Munk Laursen, Gunhild Waldemar

**Affiliations:** ^1^ Danish Dementia Research Centre, Department of Neurology Copenhagen University Hospital—Rigshospitalet Copenhagen Denmark; ^2^ National Centre for Register‐Based Research, Department of Economics and Business Economics, Aarhus BSS Aarhus University Aarhus Denmark; ^3^ Department of Clinical Medicine University of Copenhagen Copenhagen Denmark

**Keywords:** dementia, influenza, prevention, public health, vaccination

## Abstract

**Background and Purpose:**

Previous studies have reported conflicting results regarding the association between influenza vaccination and dementia. This association was investigated in a nationwide register‐based cohort study.

**Methods:**

Using nationwide registries, dementia‐free adults aged ≥65 years in Denmark from 2002 to 2018 without previous influenza vaccinations were included. Poisson regression facilitated confounder‐adjusted comparisons of dementia rates for ever versus never vaccinated, number of vaccinations and within/after 5 years from first vaccination. Sensitivity analyses included stratification on age and sex.

**Results:**

Vaccination during follow‐up was associated with a slightly higher rate of dementia when adjusted for sociodemographic factors and comorbidities, both within and after the first 5 years from first vaccination (incidence rate ratio [IRR] 1.04; 95% confidence interval [CI] 1.03–1.05). The rate of dementia decreased with increasing number of vaccinations. The highest rate was amongst those with only one vaccination (IRR 1.14; 95% CI 1.12–1.17) and the rate of dementia was only decreased amongst those with six or more vaccinations (IRR 0.95; 95% CI 0.93–0.97). Applying the same models to control outcomes of hip fracture and cancer resulted in higher rates amongst vaccinated people of 6% and 7%, respectively. Vaccinated people also had a 10% higher mortality rate.

**Discussion:**

Our results do not support the case for a preventive effect of influenza vaccination on the risk of dementia in the general population, as reported by some previous studies. However, the higher dementia rate amongst vaccinated people found in this study is probably due to residual confounding, indicated by a higher rate for control outcomes and mortality.

## Graphical abstract

In the entire Danish population of older people aged 65+, influenza vaccination was associated with a slightly higher risk of dementia. This increased risk was probably due to differences between vaccinated and unvaccinated people which could not be controlled for in the study. Unlike previous observational studies, no convincing evidence of a preventive or adverse effect of influenza vaccination on the risk of dementia was found.

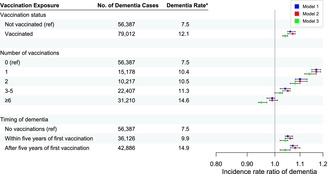

## INTRODUCTION

Dementia is a major public health challenge that globally affects an increasing number of people because of ageing populations [1]. Identifying factors that can potentially mitigate the risk of dementia is therefore of high priority [1].

It has been proposed that common adult vaccines, including the influenza vaccine, may reduce the risk of dementia [2]. Several biological mechanisms may underlie a potential protective effect of influenza vaccination. First, vaccination reduces the risk of infections in general, which have been linked to the progression of cognitive impairment and incident dementia through neuroinflammation [3, 4]. Second, influenza vaccination may reduce the risk of cardiovascular and cerebrovascular disease, which are risk factors for dementia [5, 6]. Third, in an Alzheimer's disease mouse model influenza vaccination led to an increased clearance of amyloid‐beta through microglial activation and improved performance on a memory test [7].

Six observational studies found a lower risk of dementia amongst older adults receiving influenza vaccines compared to unvaccinated persons [8, 9, 10, 11, 12, 13]. However, these studies have several limitations that may reduce their results' validity. One smaller study assessed vaccination status by self‐administered questionnaires with low reporting rates, increasing the risk of misclassification [8]. Two studies were based on selected samples of patients with chronic kidney disease [9] or chronic obstructive pulmonary disease [10], limiting the generalizability of the results. Two studies from the United States were based on large nationwide samples but restricted to veterans or selective insurance data [11, 12]; thus their results may not be generalizable to the entire population of older adults. Finally, a study from the UK was based on primary care data [13] with a questionable validity of dementia diagnosis. Furthermore, another study based on the same data found considerably higher odds of dementia amongst those vaccinated against influenza [14]. Although the latter study included a larger age span and followed the population for longer, the opposite direction of association with an almost identical data source is worrying. Thus, whether influenza vaccination is associated with the risk of dementia is still controversial and more research is needed [15].

Since 2002, all Danish citizens aged ≥65 have been offered an influenza vaccination free of charge. Therefore, the Danish registers provide a unique opportunity to investigate the association between influenza vaccination and the risk of dementia amongst older adults.

This nationwide registry‐based study investigated whether late‐life influenza vaccination, including the number and timing of vaccinations, is associated with a reduced risk of dementia.

## METHODS

### Data sources

The study is based entirely on Danish nationwide registries, enabled through linkage with a personal identifier given to all legal residents in Denmark [16].

### Population

**FIGURE 1:**
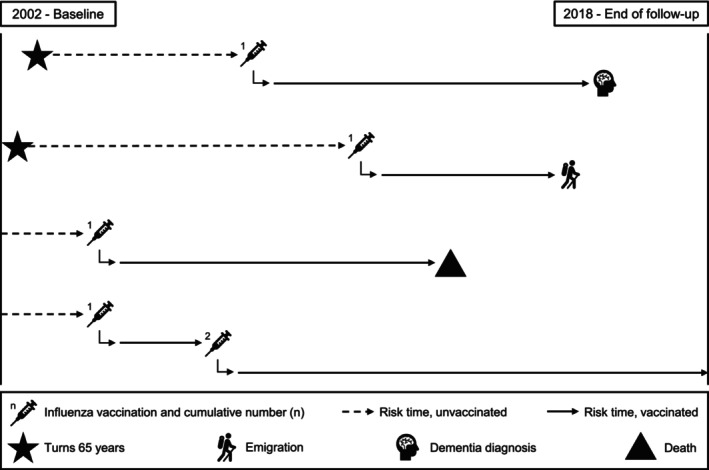
Simplified graphical representation of the study design.

From the start of the Danish influenza vaccination programme in 2002 to 2018, all Danish residents were followed from age ≥65 until dementia diagnosis, emigration or death (Figure 1). Individuals were included from 1 September 2002, or, if younger than 65 years at this time, on their 65th birthday through rolling entry. Individuals were excluded if they had been diagnosed with dementia or received an influenza vaccine before inclusion. People with mild cognitive impairment were not excluded.

### Influenza vaccinations

Since 2002, influenza vaccination has been offered free of charge to all Danish citizens ≥65 years of age, mainly in primary care. Influenza vaccination in this study was defined as a registered receipt of influenza vaccination in the Danish National Health Service Register [17] or a redeemed prescription for an influenza vaccine in the Danish National Prescription Register [18], both assessed from 2002 onwards. Available data on prescriptions redeemed before 2002 in the prescription register (from 1995) were very few and were used to exclude people with vaccinations before inclusion. Influenza vaccination was assessed in three ways: (1) ever versus never vaccinated during follow‐up; (2) number of vaccinations during follow‐up in five categories (0, 1, 2, 3–5, ≥6); and (3) the time between first influenza vaccination and dementia diagnosis defined as within or after 1, 2 and 5 years since vaccination. Individuals could contribute risk time as both unvaccinated and vaccinated depending on if/when they received their first influenza vaccination (time‐dependent variable).

### Dementia

All‐cause dementia was defined as the first registered inpatient or outpatient diagnosis of any type in the Danish National Patient Register [19] or Danish Psychiatric Central Research Register [20] or the first redeemed prescription for anti‐dementia medication in the Danish National Prescription Registry (see the Appendix, Table A1, for details). Anti‐dementia medication included donepezil, galantamine, rivastigmine and memantine.

### Covariates

The covariates in this study were selected based on their potential for confounding the association between influenza vaccination and dementia, that is, factors associated with receiving an influenza vaccination and independent risk factors of dementia. Age, sex and marital status were identified through the Civil Registration System [21], educational attainment through the Danish education registers [22] and comorbidities through the hospital and prescription registries mentioned above. Age and calendar year were the underlying time scales in our statistical model and were included as time‐dependent covariates. Sex and educational attainment were assessed at the start of follow‐up. Educational attainment was divided into four categories: high (short‐term further education, bachelor's degree, research degree; ≥12 years), medium (upper secondary, business high school, vocational internship; 10–12 years), low (primary school; ≤9 years) and unknown. Marital status was included as a time‐dependent covariate, allowing for multiple changes throughout the study period. Information on marital status was available at the end of each year without exact marriage dates. Therefore, the status date was approximated as 1 January of the following year. The following comorbidities were chosen based on their biological plausibility of being risk factors for dementia: stroke, cardiac arrhythmia, peripheral vascular disease, hypertension, diabetes, hyperlipidaemia, depression, alcohol use and Parkinson's disease. Comorbidities were identified through the first instance of an inpatient or outpatient diagnosis in the hospital registers or the first redeemed prescription in the prescription register if followed by a second redeemed prescription within the following year. Medication was only used to identify comorbidities if the drugs were specific to treating those comorbidities (see Table A1 for the specific diagnostic and medication codes used in this study). Comorbidities were included as time‐dependent variables, persisting from first identification onwards during the remaining follow‐up period.

### Statistical analyses

In the main analyses, the incidence rate ratios (IRRs) by vaccine status for all‐cause dementia were estimated by log‐linear Poisson regression with the logarithm of the person‐years at risk as an offset variable. The Poisson model requires rates to be piecewise constant within each time interval. In this study, the underlying time scales were age and calendar time, both in 1‐year groups. This method is equivalent to a Cox regression survival model [23, 24]. Compared with no vaccination during the study period, the effect of any vaccination at age ≥65, the number of vaccinations and the time between the first vaccination and dementia diagnosis were estimated in the following models. In model 1, adjustments were made for age, sex and calendar year. In model 2, marital status and educational attainment were additionally adjusted for. In model 3, further adjustments were made for the comorbidities mentioned above.

Several sensitivity analyses were performed to explore the validity of our main results. First, whether the results could have been influenced by confounding was explored. This was done by testing whether the rates of cancer and hip fractures, that is, negative control outcomes [25] assumed to be causally unrelated to the exposure, were different for vaccinated and unvaccinated individuals. Second, whether the competing risk of death could impact our results was explored by estimating the mortality rate ratio for vaccinated compared to unvaccinated. Third, the main association was analysed in subgroups stratified by 10‐year age intervals (65–74, 75–84 and ≥85), by three calendar periods (2002–2007, 2008–2013 and 2014–2018) and by sex. Fourth, older adults living in the municipality of Copenhagen have been offered free influenza vaccines since 1996 outside the national programme that were not registered in any of our available data sources. Thus, an unknown number of inhabitants of Copenhagen may be misclassified as unvaccinated during our study period. To examine the impact of this misclassification, IRRs were estimated in a subpopulation without individuals living in Copenhagen who were eligible to receive the free influenza vaccination offer. Of those, individuals living in Copenhagen at any time between 1996 and inclusion into our study were excluded. After inclusion individuals were censored on the date they moved to Copenhagen. SAS version 9.4 was used for all statistical analyses. Figure 3 was created using R version 4.2.2.

## RESULTS

**FIGURE 2:**
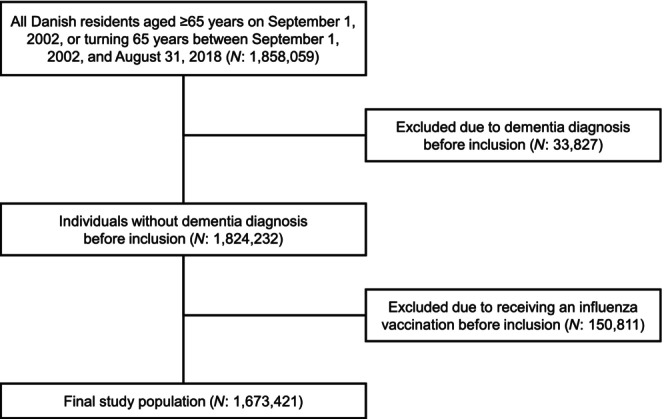
Selection of study population. Inclusion was 1 September 2002 or when subsequently turning 65 years old.

During the total follow‐up of 14,065,154 person‐years, 135,399 were diagnosed with dementia, resulting in a crude incidence rate of 9.6 per 1000 person‐years. Figure 2 shows how the study population was selected.

Table 1 shows the distribution of covariates in the study population stratified by vaccination status, presented both as number of individuals at inclusion and as person‐years during the study period. At inclusion, people vaccinated during follow‐up had higher educational attainment and were more often diagnosed with hypertension and hyperlipidaemia, but less often diagnosed with alcohol abuse and Parkinson's disease. In contrast, vaccinated people had a slightly higher contribution of person time from females compared to unvaccinated and vaccinated people also had considerably more person time recorded with hypertension, hyperlipidaemia, cardiac arrhythmia and diabetes. Finally, vaccinated people were slightly older at dementia diagnosis.

**TABLE 1:** Study population characteristics stratified by vaccination status.

	Vaccinated		Not vaccinated	
Characteristics	Number of people: 991,729^a^	Person‐years: 6,549,905^b^	Number of people: 681,692^a^	Person‐years: 7,515,249^b^
Sex (*N*, %)				
Female	531,928 (53.6)	3,647,297 (55.7)	366,725 (53.8)	4,123,719 (54.9)
Educational attainment (*N*, %)^c^				
High	46,443 (4.7)	271,197 (4.1)	25,559 (3.7)	294,269 (3.9)
Medium	142,030 (14.3)	875,476 (13.4)	87,518 (12.8)	983,649 (13.1)
Low	716,772 (72.3)	4,968,884 (75,9)	476,994 (70.0)	5,659,738 (75.3)
Unknown	86,484 (8.7)	434,348 (6.6)	91,621 (13.4)	577,593 (7.7)
Marital status (*N*, %)^c^				
Married	645,012 (65.0)	3,589,316 (54.8)	380,909 (55.9)	4,267,797 (56.8)
Unmarried	343,720 (34.7)	2,960,337 (45.2)	296,090 (43.4)	3,217,893 (42.8)
Unknown	2997 (0.3)	252 (0.0)	4693 (0.7)	29,559 (0.4)
Comorbidities (*N*, %)^c^				
Hypertension	451,693 (45.5)	4,473,372 (68.3)	280,922 (41.2)	3,751,639 (49.9)
Hyperlipidaemia	161,933 (16.3)	2,640,546 (40.3)	96,670 (14.2)	1,778,205 (23.7)
Peripheral vascular disease	6397 (0.6)	37,380 (0.6)	4646 (0.7)	35,640 (0.5)
Stroke	8070 (0.8)	49,677 (0.8)	6426 (0.9)	50,521 (0.7)
Cardiac arrhythmia	12,903 (1.3)	149,175 (2.3)	7586 (1.1)	86,333 (1.1)
Diabetes	59,617 (6.0)	768,863 (11.7)	40,517 (5.9)	531,831 (7.1)
Parkinson's disease	10,053 (1.0)	177,460 (2.7)	9306 (1.4)	111,491 (1.5)
Depression	17,384 (1.8)	115,987 (1.8)	11,487 (1.7)	117,803 (1.6)
Alcohol abuse	14,456 (1.5)	102,598 (1.6)	13,924 (2.0)	119,726 (1.6)
Age at dementia diagnosis (median, Q1, Q2)	82.9 (78.1, 87.4)	–	81.6 (75.7, 86.6)	–

^a^ At inclusion.

^b^ During follow‐up.

^c^ At inclusion, that is, 1 September 2002 or when subsequently turning 65 years old.

**FIGURE 3:**
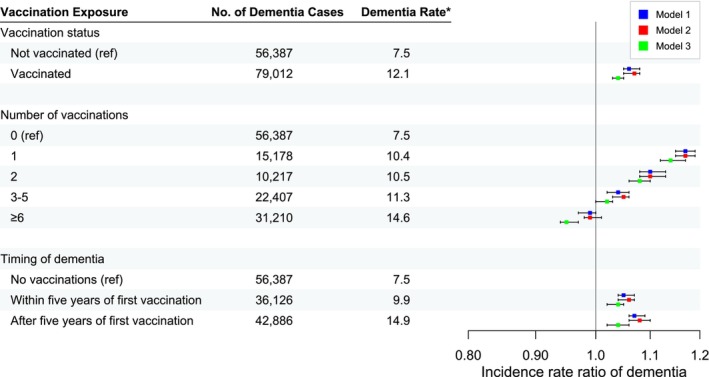
Incidence rate ratios of dementia according to different influenza vaccine exposures. The *x*‐axis in the graph has been log‐transformed. *Crude incidence rate of dementia per 1000 person‐years. Model 1: Adjustment for age, calendar year and sex. Model 2: Model 1 + adjustment for marital status and educational attainment. Model 3: Model 2 + adjustment for comorbidities.

Figure 3 shows the IRRs of dementia and related 95% confidence intervals (CIs), number of dementia cases during follow‐up, and crude incidence rate of dementia for ever versus never vaccinated, number of vaccinations, and within or after 5 years from the first vaccination. A 4% higher incidence rate of dementia was found amongst ever vaccinated compared with never vaccinated in a fully adjusted model (IRR 1.04; 95% CI 1.02–1.05) (see Table 2). Compared to no vaccination, one, two or three to five vaccinations were associated with a slightly higher dementia rate whilst those with six or more vaccinations had the only lower rate. The incidence rate was 4% higher within and after 5 years since the first vaccination compared to no vaccinations (Table A2). The rate was similarly increased within and after 1 year since the first vaccination, 6% and 3% respectively. The rate was also similar within and after 2 years since the first vaccination, 5% and 3% respectively (data not shown).

**TABLE 2:** Incidence rate ratio for vaccinated people compared to those not vaccinated.

	Model 1^a^		Model 2^b^		Model 3^c^	
	IRR	95% CI	IRR	95% CI	IRR	95% CI
Dementia as the outcome						
Not vaccinated (ref.)	1		1		1	
Vaccinated	1.06	1.05–1.08	1.07	1.05–1.08	1.04	1.03–1.05
Death as the outcome						
Not vaccinated (ref.)	1		1		1	
Vaccinated	1.14	1.13–1.14	1.16	1.16–1.17	1.10	1.09–1.10
Cancer as the outcome						
Not vaccinated (ref.)	1		1		1	
Vaccinated	1.08	1.07–1.09	1.08	1.07–1.09	1.07	1.06–1.07
Hip fracture as the outcome						
Not vaccinated (ref.)	1		1		1	
Vaccinated	1.07	1.04–1.11	1.08	1.05–1.12	1.06	1.03–1.10

Abbreviations: CI, confidence interval; IRR, incidence rate ratio.

^a^ Adjustment for age, calendar year and sex.

^b^ Model 1 + adjustment for marital status and educational attainment.

^c^ Model 2 + adjustment for comorbidities.

The negative control outcome analyses showed almost identical results to the main analyses, with a 7% higher cancer rate and a 6% higher hip fracture rate amongst those with a recorded influenza vaccination. Vaccinated people also had a 10% higher mortality rate compared with unvaccinated in the fully adjusted model (see Tables 2 and A3, A4, A5).

There was an inverse relation between age group and dementia rate amongst vaccinated versus unvaccinated people: in ages 65–74, the dementia rate was 11% higher for vaccinated, 5% higher in ages 75–84 and 3% lower in ages ≥85 (see Table A6). The dementia rate was 10% lower for vaccinated people during the early calendar period from 2002 to 2007, 7% higher for 2008–2013 and 18% higher for 2014–2018 (see Table A7). No considerable differences were found between the sex‐stratified and main analyses (data not shown).

Excluding inhabitants of Copenhagen decreased the total person‐years at risk and the number of dementia cases by approximately 6% and 10%, respectively (data not shown). The IRRs of dementia, mortality and control outcomes were similar or identical in this subpopulation to those of the entire study population (see Tables A8, A9, A10, A11, A12, A13).

## DISCUSSION

In this nationwide retrospective cohort study, a 4% higher dementia rate associated with influenza vaccination amongst older adults was found when adjusting for relevant demographic and health‐related confounders. The causality of this finding, however, is questioned by the results of several of our additional analyses. First, it aligns poorly with the complex dose–response relationship between number of vaccinations and dementia rate, with a 14% higher dementia rate after one vaccination and gradually decreasing until it was 5% lower after six vaccinations. Second, the similarity of the main analyses and associations with the control outcomes of hip fracture and cancer, as well as the higher mortality rate amongst vaccinated people, indicate considerable residual confounding due to differences in health status and health‐seeking behaviour. Third, the difference in the association between vaccination and dementia across age groups and time period may suggest bias due to difference in uptake patterns across age and since the start of the Danish vaccination programme.

Our findings did not align with any previous study in this area. In all but one of the previous studies, influenza vaccination was associated with a reduction of the dementia risk of between 40% [12] and 4% [13]. The 39% higher odds of dementia amongst vaccinated people in one study [14] far exceeded the rate differences in the present study. The estimated associations in both the present and previous studies may have been influenced by bias to an extent that it affected the interpretation and perspectives of the findings. Whilst it can be challenging to predict the direction and magnitude of bias in all observational research, some sources of potential bias in the present and previous studies can be identified. As Douros et al. remark, prodromal dementia symptoms may lower the likelihood of vaccination, potentially introducing protopathic bias (i.e., reverse causality) which could mask a higher dementia rate amongst vaccinated people [14]. Conversely, detection bias may have increased the dementia rate amongst vaccinated people if general practitioner visits for vaccination lead to a higher likelihood of being diagnosed with dementia. No strong indications of the presence of these bias mechanisms were found in the present study, as there was no difference in dementia rate within or after the first year, first 2 years or first 5 years after the first vaccination. None of the studies finding a lower dementia rate amongst vaccinated people examined the presence of reverse causality or detection bias in their data.

Our study has several strengths. First, the Danish national registries contain highly valid information on influenza vaccination [17], dementia diagnosis [26], comorbidities [19] and sociodemographic factors [21, 22] for the entire Danish population of older adults. Second, the registries allowed for a long follow‐up period with a lower mean age at cohort entry (65 years) compared with many of the previous studies. Third, the large sample facilitated precise estimation of associations in relevant subgroups and exploration of whether uncontrolled confounding [25] or competing risk of death could have influenced our findings. Finally, the cohort design allowed us to account for time‐dependent confounding. There are also several limitations to our study. As mentioned above, the results of our negative control outcome analyses suggest that uncontrolled differences in health and behaviour may have biased our findings [25]. Furthermore, there is also a risk of information bias when using data from administrative registers to investigate potential health effects, and the present study is no different. First, underdiagnosis of dementia in healthcare registries is a common issue across European populations, including the Danish [27]. To mitigate the potential issue of misclassification of dementia, cases from all available sources in the Danish registries, that is, data on secondary care diagnoses and prescription medication, were identified. Despite these efforts, misclassification of dementia, especially if differential, may still have impacted our results. Second, misclassification of exposure to influenza vaccines is also possible. It is known that older adults living in Copenhagen were offered free vaccines that were not registered in the national registers for primary care services or redeemed prescriptions. However, excluding inhabitants of Copenhagen from our study population had minor to no impact on the crude and adjusted associations between vaccination and dementia, death or the control outcomes. Furthermore, vaccinations through self‐payment or place of employment could not be identified through available national registers. However, as the entire study population was eligible for free vaccinations during the study period, combined with the widespread availability of influenza vaccines across Denmark, self‐paid vaccination is not expected to be common enough to have affected the overall findings. Third, for a considerable proportion of the study population, educational attainment could not be assessed, and for a very small proportion marital status was missing. The proportion with an unknown educational level (and marital status) was higher amongst unvaccinated than vaccinated people, potentially biasing the adjusted effect estimates. However, the bias is probably negligible, as adjusting for the two variables had no meaningful impact on the estimates. Fourth, as exact marriage dates were not available, marital status may have been misclassified for a limited period in a few individuals. However, it is not likely that this potential misclassification has affected the results. Finally, identifying comorbidities using registry data may also include a risk of misclassification. Validation studies exist for the majority of, but not all, codes used in the identification of comorbidities in the present study [28, 29, 30, 31, 32, 33], generally showing moderate to high validity (see the Appendix for details of the specific codes). As mentioned above, differences in the average health status for vaccinated compared to unvaccinated people may persist despite adjustment for relevant comorbidities. On the one hand, vaccinated people may be frailer than those without any vaccinations during the study period, in ways that could not be adjusted for in the present study [34]. This may have contributed to the higher dementia and mortality rate when comparing ever versus never vaccinated. On the other hand, those vaccinated regularly each year may have a higher health status than those with few or no vaccinations [35]. This may explain why lower rates of dementia and death were found amongst those with six or more vaccinations compared with unvaccinated, but a higher rate amongst those with fewer than six vaccinations. Future studies should consider additional and valid measures for healthcare‐seeking behaviour and healthcare utilization to further explore the impact of residual confounding/healthy vaccinee effect. The cohort design did not allow for a lag period between vaccination and dementia diagnosis as there was no index date for those without dementia. Therefore, to investigate the potential impact of reverse causality (i.e., protopathic and detection bias), the dementia rates within 5 years and after 5 years of the first vaccination were estimated [28, 29, 30, 31, 32, 33].

The evidence taken together does not provide a convincing case for a protective effect of influenza vaccination on the risk of dementia. However, based on our study there is no obvious reason to warn against a potential increased risk of dementia related to influenza vaccination. Further observational studies should focus on minimizing confounding from health behaviour and health status as well as on investigating whether preventive effects could exist for specific subpopulations and vaccination at earlier ages than previously studied [15]. More research is also needed to elucidate the central nervous system and immune system effects of common adult vaccinations.

## AUTHOR CONTRIBUTIONS

**Andreas Moses Appel:** Conceptualization; funding acquisition; writing – original draft; methodology; writing – review and editing; investigation; formal analysis; project administration. **Janet Janbek:** Conceptualization; investigation; writing – review and editing; methodology; formal analysis; supervision. **Christina Jensen‐Dahm:** Conceptualization; investigation; writing – review and editing; methodology; supervision. **Thomas Munk Laursen:** Writing – review and editing; methodology; validation; formal analysis; supervision. **Gunhild Waldemar:** Conceptualization; investigation; funding acquisition; writing – review and editing; methodology; supervision.

## FUNDING INFORMATION

The Danish Ministry of Health supports the Danish Dementia Research Centre. This study was further supported by Alzheimer‐forskningsfonden. None of the funders was involved in any phase of the study.

## CONFLICT OF INTEREST STATEMENT

The authors declare no conflicts of interest.

## ETHICS STATEMENT

Danish law does not require informed patient consent or ethics committee approval for registry‐based studies [36]. This study is part of a project that has been approved by the Danish Data Protection Agency, Statistics Denmark and the Danish Health Data Authority.

## Data Availability

This study is based entirely on anonymized personal data in the Danish national registries. These data are only accessible with authorization from the Danish Data Protection Agency, the Danish Health Authority, the Danish Health Data Authority, the Danish Medicines Authority and Statistics Denmark.

## Appendix

**TABLE A1:** Codes in the International Classification of Diseases (ICD) and Anatomical Therapeutic Chemical Classification System (ATC) for identification of dementia and comorbidities.

	ICD‐8	ICD‐10	ATC codes
Dementia type			
Alzheimer's disease	290.10	F00.0–00.9, G30.0–G30.9	N06DA02–N06DA04, N06DA52, N06DA53, N06DX01
Vascular dementia	293.09–19	F01.0–01.9	
Frontotemporal dementia	290.11	F02.0, G31.0A, G31.0B	
Dementia without specification	290.09–19	F03.9, G31.9	
Other dementias	–	G31.8, G31.8E, F02.1–F02.8	
Comorbidities^a^, ^b^			
Hypertension	400–404, 410.09, 411.09, 412.09, 413.09, 414.09, 435.09, 437.00–437.09, 438.09	*I10*–*I13, I15*	*C02*–*C04, C07*–*C09*
Hyperlipidaemia	279.00	*E78*	C10
Peripheral vascular disease	440–445	I70, *I71*, I72, I73, I74, I77	
Stroke	430, 431, 433, 434, 436	*I60*–*I64, I69*	
Cardiac arrhythmia	427.93, 427.94, 427.97	*I48, I49*	C01B
Diabetes	249, 250	*E10*–*E14*	A10A, A10B
Parkinson's disease	66, 342.99	*G20*	N04
Depression	296.09, 296.29, 296.99, 298.09, 300.4, 300.19	*F32*	
Alcohol use	303.09, 303.19, 303.20, 303.28, 303.29, 303.99	F10.1	

^a^ ATC codes were registered at least twice within a 12‐month period.

^b^ ICD and ATC codes in italics are validated in the Danish registries (see references in the main text for validation studies).

**TABLE A2:** Incidence rate ratio of all‐cause dementia by any vaccination, number and timing of vaccination, entire study population.

	Model 1^a^		Model 2^b^		Model 3^c^	
	IRR	95% CI	IRR	95% CI	IRR	95% CI
Ever versus never vaccinated						
Not vaccinated (ref.)	1		1		1	
Vaccinated	1.06	1.05–1.08	1.07	1.05–1.08	1.04	1.03–1.05
No. of vaccinations						
0 (ref.)	1		1		1	
1	1.17	1.15–1.19	1.17	1.15–1.19	1.14	1.12–1.17
2	1.10	1.08–1.13	1.10	1.08–1.13	1.08	1.06–1.10
3–5	1.04	1.02–1.06	1.05	1.03–1.06	1.02	1.00–1.03
≥6	0.99	0.97–1.00	0.99	0.98–1.01	0.95	0.94–0.97
Timing of dementia						
No vaccination (ref)	1		1		1	
Within 5 years	1.05	1.04–1.07	1.06	1.04–1.07	1.04	1.02–1.05
After 5 years	1.07	1.06–1.09	1.08	1.06–1.10	1.04	1.02–1.06

Abbreviations: CI, confidence interval; IRR, incidence rate ratio.

^a^ Adjustment for age, calendar year and sex.

^b^ Model 1 + adjustment for marital status and educational attainment.

^c^ Model 2 + adjustment for comorbidities.

**TABLE A3:** Incidence rate ratio of all‐cause mortality by any vaccination, number and timing of vaccination, entire study population.

	Model 1^a^		Model 2^b^		Model 3^c^	
	IRR	95% CI	IRR	95% CI	IRR	95% CI
Ever versus never vaccinated						
Not vaccinated (ref.)	1		1		1	
Vaccinated	1.14	1.13–1.14	1.16	1.16–1.17	1.10	1.09–1.10
No. of vaccinations						
0 (ref.)	1		1		1	
1	1.20	1.19–1.21	1.22	1.21–1.23	1.16	1.15–1.17
2	1.14	1.13–1.16	1.17	1.16–1.18	1.11	1.10–1.12
3–5	1.11	1.10–1.12	1.14	1.13–1.15	1.08	1.07–1.08
≥6	1.10	1.09–1.11	1.13	1.12–1.14	1.05	1.05–1.06
Timing of dementia						
No vaccination (ref.)	1		1		1	
Within 5 years	1.11	1.11–1.12	1.15	1.14–1.15	1.09	1.08–1.09
After 5 years	1.17	1.16–1.18	1.20	1.19–1.20	1.11	1.11–1.12

Abbreviations: CI, confidence interval; IRR, incidence rate ratio.

^a^ Adjustment for age, calendar year and sex.

^b^ Model 1 + adjustment for marital status and educational attainment.

^c^ Model 2 + adjustment for comorbidities.

**TABLE A4:** Incidence rate ratio of cancer by any vaccination, number and timing of vaccination, entire study population.

	Model 1^a^		Model 2^b^		Model 3^c^	
	IRR	95% CI	IRR	95% CI	IRR	95% CI
Ever versus never vaccinated						
Not vaccinated (ref.)	1		1		1	
Vaccinated	1.08	1.07–1.09	1.08	1.07–1.09	1.07	1.06–1.07
No. of vaccinations						
0 (ref.)	1		1		1	
1	1.08	1.07–1.09	1.08	1.07–1.09	1.07	1.05–1.08
2	1.07	1.06–1.09	1.07	1.06–1.09	1.06	1.04–1.07
3–5	1.07	1.06–1.09	1.07	1.06–1.08	1.06	1.04–1.07
≥6	1.10	1.09–1.12	1.10	1.09–1.11	1.08	1.07–1.10
Timing of dementia						
No vaccination (ref.)	1		1		1	
Within 5 years	1.08	1.07–1.09	1.08	1.07–1.09	1.06	1.05–1.07
After 5 years	1.09	1.08–1.10	1.09	1.08–1.10	1.07	1.06–1.08

Abbreviations: CI, confidence interval; IRR, incidence rate ratio.

^a^ Adjustment for age, calendar year and sex.

^b^ Model 1 + adjustment for marital status and educational attainment.

^c^ Model 2 + adjustment for comorbidities.

**TABLE A5:** Incidence rate ratio of hip fracture by any vaccination, number and timing of vaccination, entire study population.

	Model 1^a^		Model 2^b^		Model 3^c^	
	IRR	95% CI	IRR	95% CI	IRR	95% CI
Ever versus never vaccinated						
Not vaccinated (ref.)	1		1		1	
Vaccinated	1.07	1.04–1.11	1.08	1.05–1.12	1.06	1.03–1.10
No. of vaccinations						
0 (ref.)	1		1		1	
1	1.08	1.03–1.14	1.09	1.03–1.14	1.07	1.01–1.12
2	1.15	1.09–1.22	1.16	1.09–1.23	1.14	1.07–1.21
3–5	1.04	0.99–1.08	1.04	1.00–1.08	1.02	0.98–1.07
≥6	1.06	1.01–1.11	1.08	1.01–1.13	1.05	1.01–1.10
Timing of dementia						
No vaccination (ref.)	1		1		1	
Within 5 years	1.06	1.02–1.10	1.06	1.02–1.11	1.05	1.01–1.09
After 5 years	1.09	1.05–1.14	1.11	1.06–1.16	1.08	1.04–1.13

Abbreviations: CI, confidence interval; IRR, incidence rate ratio.

^a^ Adjustment for age, calendar year and sex.

^b^ Model 1 + adjustment for marital status and educational attainment.

^c^ Model 2 + adjustment for comorbidities.

**TABLE A6:** Incidence rate ratio of dementia for vaccinated compared to those not vaccinated in 10‐year age groups, entire study population.

	Model 1^a^		Model 2^b^		Model 3^c^	
	IRR	95% CI	IRR	95% CI	IRR	95% CI
Age 65–74 years						
Not vaccinated (ref.)	1		1		1	
Vaccinated	1.20	1.16–1.23	1.22	1.18–1.25	1.11	1.08–1.14
Age 75–84 years						
Not vaccinated (ref.)	1		1		1	
Vaccinated	1.08	1.06–1.10	1.08	1.07–1.10	1.05	1.03–1.07
Age 85+ years						
Not vaccinated (ref.)	1		1		1	
Vaccinated	0.97	0.95–0.99	0.97	0.95–0.99	0.97	0.95–0.99

Abbreviations: CI, confidence interval; IRR, incidence rate ratio.

^a^ Adjustment for age, calendar year and sex.

^b^ Model 1 + adjustment for marital status and educational attainment.

^c^ Model 2 + adjustment for comorbidities.

**TABLE A7:** Incidence rate ratio of dementia for vaccinated compared to those not vaccinated in calendar periods, entire study population.

	Model 1^a^		Model 2^b^		Model 3^c^	
	IRR	95% CI	IRR	95% CI	IRR	95% CI
2002–2007						
Not vaccinated (ref.)	1		1		1	
Vaccinated	0.90	0.89–0.92	0.91	0.89–0.93	0.90	0.88–0.92
2008–2013						
Not vaccinated (ref.)	1		1		1	
Vaccinated	1.09	1.07–1.11	1.10	1.08–1.12	1.07	1.05–1.09
2014–2018						
Not vaccinated (ref.)	1		1		1	
Vaccinated	1.23	1.20–1.26	1.24	1.21–1.27	1.18	1.16–1.21

Abbreviations: CI, confidence interval; IRR, incidence rate ratio.

^a^ Adjustment for age, calendar year and sex.

^b^ Model 1 + adjustment for marital status and educational attainment.

^c^ Model 2 + adjustment for comorbidities.

**TABLE A8:** Incidence rate ratio of all‐cause dementia by any vaccination, number and timing of vaccination, without inhabitants of the municipality of Copenhagen.

	Model 1^a^		Model 2^b^		Model 3^c^	
	IRR	95% CI	IRR	95% CI	IRR	95% CI
Ever versus never vaccinated						
Not vaccinated (ref.)	1		1		1	
Vaccinated	1.10	1.09–1.12	1.10	1.09–1.12	1.07	1.06–1.09
No. of vaccinations						
0 (ref.)	1		1		1	
1	1.20	1.17–1.22	1.20	1.18–1.22	1.17	1.15–1.20
2	1.14	1.12–1.17	1.15	1.12–1.17	1.12	1.09–1.14
3–5	1.08	1.06–1.10	1.08	1.06–1.10	1.05	1.03–1.07
≥6	1.03	1.01–1.05	1.04	1.02–1.05	0.99	0.98–1.01
Timing of dementia						
No vaccination (ref.)	1		1		1	
Within 5 years	1.09	1.07–1.11	1.09	1.08–1.11	1.07	1.05–1.08
After 5 years	1.12	1.10–1.14	1.12	1.10–1.14	1.08	1.06–1.09

Abbreviations: CI, confidence interval; IRR, incidence rate ratio.

^a^ Adjustment for age, calendar year and sex.

^b^ Model 1 + adjustment for marital status and educational attainment.

^c^ Model 2 + adjustment for comorbidities.

**TABLE A9:** Incidence rate ratio of all‐cause mortality by any vaccination, number and timing of vaccination, without inhabitants of the municipality of Copenhagen.

	Model 1^a^		Model 2^b^		Model 3^c^	
	IRR	95% CI	IRR	95% CI	IRR	95% CI
Ever versus never vaccinated						
Not vaccinated (ref.)	1		1		1	
Vaccinated	1.16	1.15–1.17	1.19	1.18–1.20	1.12	1.11–1.13
No. of vaccinations						
0 (ref.)	1		1		1	
1	1.22	1.21–1.23	1.24	1.23–1.25	1.18	1.17–1.19
2	1.17	1.16–1.18	1.20	1.18–1.21	1.13	1.12–1.14
3–5	1.14	1.13–1.15	1.17	1.16–1.18	1.10	1.09–1.11
≥6	1.12	1.11–1.13	1.16	1.15–1.17	1.08	1.07–1.08
Timing of dementia						
No vaccination (ref.)	1		1		1	
Within 5 years	1.14	1.13–1.15	1.17	1.16–1.18	1.11	1.10–1.12
After 5 years	1.19	1.28–1.20	1.22	1.21–1.23	1.13	1.13–1.14

Abbreviations: CI, confidence interval; IRR, incidence rate ratio.

^a^ Adjustment for age, calendar year and sex.

^b^ Model 1 + adjustment for marital status and educational attainment.

^c^ Model 2 + adjustment for comorbidities.

**TABLE A10:** Incidence rate ratio of cancer by any vaccination, number and timing of vaccination, without inhabitants of the municipality of Copenhagen.

	Model 1^a^		Model 2^b^		Model 3^c^	
	IRR	95% CI	IRR	95% CI	IRR	95% CI
Ever versus never vaccinated						
Not vaccinated (ref.)	1		1		1	
Vaccinated	1.10	1.09–1.11	1.09	1.09–1.10	1.08	1.07–1.09
No. of vaccinations						
0 (ref.)	1		1		1	
1	1.09	1.08–1.11	1.09	1.08–1.10	1.08	1.06–1.09
2	1.09	1.07–1.10	1.09	1.07–1.10	1.07	1.05–1.09
3–5	1.09	1.08–1.10	1.09	1.07–1.10	1.07	1.06–1.08
≥6	1.12	1.10–1.13	1.11	1.10–1.12	1.09	1.08–1.11
Timing of dementia						
No vaccination (ref.)	1		1		1	
Within 5 years	1.09	1.08–1.10	1.09	1.08–1.10	1.07	1.06–1.08
After 5 years	1.11	1.09–1.12	1.10	1.09–1.12	1.08	1.07–1.10

Abbreviations: CI, confidence interval; IRR, incidence rate ratio.

^a^ Adjustment for age, calendar year and sex.

^b^ Model 1 + adjustment for marital status and educational attainment.

^c^ Model 2 + adjustment for comorbidities.

**TABLE A11:** Incidence rate ratio of hip fracture by any vaccination, number and timing of vaccination, without inhabitants of the municipality of Copenhagen.

	Model 1^a^		Model 2^b^		Model 3^c^	
	IRR	95% CI	IRR	95% CI	IRR	95% CI
Ever versus never vaccinated						
Not vaccinated (ref.)	1		1		1	
Vaccinated	1.09	1.05–1.13	1.10	1.06–1.14	1.07	1.03–1.11
No. of vaccinations						
0 (ref.)	1		1		1	
1	1.08	1.02–1.14	1.08	1.02–1.15	1.06	1.00–1.12
2	1.16	1.08–1.24	1.17	1.09–1.25	1.14	1.06–1.21
3–5	1.06	1.01–1.12	1.07	1.01–1.13	1.04	0.99–1.09
≥6	1.09	1.04–1.15	1.11	1.06–1.17	1.08	1.02–1.13
Timing of dementia						
No vaccination (ref.)	1		1		1	
Within 5 years	1.07	1.02–1.12	1.08	1.03–1.13	1.05	1.01–1.10
After 5 years	1.12	1.07–1.17	1.13	1.08–1.18	1.09	1.04–1.14

Abbreviations: CI, confidence interval; IRR, incidence rate ratio.

^a^ Adjustment for age, calendar year and sex.

^b^ Model 1 + adjustment for marital status and educational attainment.

^c^ Model 2 + adjustment for comorbidities.

**TABLE A12:** Incidence rate ratio of dementia for vaccinated compared to those not vaccinated in 10‐year age groups, without inhabitants of the municipality of Copenhagen.

	Model 1^a^		Model 2^b^		Model 3^c^	
	IRR	95% CI	IRR	95% CI	IRR	95% CI
Age 65–74 years						
Not vaccinated (ref.)	1		1		1	
Vaccinated	1.22	1.18–1.25	1.24	1.20–1.27	1.12	1.09–1.16
Age 75–84 years						
Not vaccinated (ref.)	1		1		1	
Vaccinated	1.11	1.08–1.13	1.11	1.09–1.13	1.08	1.06–1.10
Age 85+ years						
Not vaccinated (ref.)	1		1		1	
Vaccinated	1.02	1.00–1.05	1.02	1.00–1.04	1.02	1.00–1.04

Abbreviations: CI, confidence interval; IRR, incidence rate ratio.

^a^ Adjustment for age, calendar year and sex.

^b^ Model 1 + adjustment for marital status and educational attainment.

^c^ Model 2 + adjustment for comorbidities.

**TABLE A13:** Incidence rate ratio of dementia for vaccinated compared to those not vaccinated in calendar periods, without inhabitants of the municipality of Copenhagen.

	Model 1^a^		Model 2^b^		Model 3^c^	
	IRR	95% CI	IRR	95% CI	IRR	95% CI
2002–2007						
Not vaccinated (ref.)	1		1		1	
Vaccinated	0.94	0.92–0.97	0.95	0.92–0.97	0.94	0.91–0.96
2008–2013						
Not vaccinated (ref.)	1		1		1	
Vaccinated	1.15	1.12–1.17	1.16	1.13–1.18	1.12	1.10–1.14
2014–2018						
Not vaccinated (ref.)s	1		1		1	
Vaccinated	1.24	1.21–1.27	1.24	1.21–1.28	1.19	1.16–1.22

Abbreviations: CI, confidence interval; IRR, incidence rate ratio.

^a^ Adjustment for age, calendar year and sex.

^b^ Model 1 + adjustment for marital status and educational attainment.

^c^ Model 2 + adjustment for comorbidities.
